# Controlling Shear Rate for Designable Thermal Conductivity in Direct Ink Printing of Polydimethylsiloxane/Boron Nitride Composites

**DOI:** 10.3390/polym15163489

**Published:** 2023-08-21

**Authors:** Bing Xiao, Xinmei Zheng, Yang Zhao, Bingxue Huang, Pan He, Biyou Peng, Gang Chen

**Affiliations:** 1 School of Materials Science and Engineering, Xihua University, Chengdu 610039, China; scxiaobing@163.com (B.X.); xinmeizheng0830@163.com (X.Z.); 18140238692@163.com (Y.Z.); pengbiyou@126.com (B.P.); 2 Sichuan Provincial Engineering Research Center of Functional Development and Application of High Performance Special Textile Materials, Chengdu Textile College, Chengdu 611731, China; hepan-2009@163.com; 3 Sichuan Province Engineering Technology Research Center of Powder Metallurgy, Chengdu University, Chengdu 610106, China

**Keywords:** 3D printing, elastomers, polydimethylsiloxane, thermal conductivity, shear rate

## Abstract

Efficient heat dissipation is vital for advancing device integration and high-frequency performance. Three-dimensional printing, famous for its convenience and structural controllability, facilitates complex parts with high thermal conductivity. Despite this, few studies have considered the influence of shear rate on the thermal conductivity of printed parts. Herein, polydimethylsiloxane/boron nitride (PDMS/BN) composites were prepared and printed by direct ink writing (DIW). In order to ensure the smooth extrusion of the printing process and the structural stability of the part, a system with 40 wt% BN was selected according to the rheological properties. In addition, the effect of printing speed on the morphology of BN particles during 3D printing was studied by XRD, SEM observation, as well as ANSYS Polyflow simulation. The results demonstrated that increasing the printing speed from 10 mm/s to 120 mm/s altered the orientation angle of BN particles from 78.3° to 35.7°, promoting their alignment along the printing direction due to the high shear rate experienced. The resulting printed parts accordingly exhibited an impressive thermal conductivity of 0.849 W∙m^−1^∙K^−1^, higher than the 0.454 W∙m^−1^∙K^−1^ of the control sample. This study provides valuable insights and an important reference for future developments in the fabrication of thermal management devices with customizable thermal conductivity.

## 1. Introduction

The rapid development of technology, especially 5G and microchip technology, has led to a shift toward higher frequency and integration levels in electronic devices [1,2]. However, this evolution also brings new challenges, especially in terms of thermal management. Due to the operation and miniaturization of high-frequency devices, a large amount of capacity is quickly accumulated in a limited space, potentially leading to overheating, which could compromise their performance and even lifespan [3,4]. Therefore, the application of high-performance thermal management materials (TMM) is extremely important, as they can absorb, disperse, and accelerate the heat generated by the device to maintain its normal operation [5]. As for highly integrated and high-frequency devices, this demand is more urgent since these devices generate a large amount of heat in a short time [6]. Facing severe thermal management challenges urgently, the design and fabrication of high-performance thermal management materials (TMMs) have been attracting great attention.

Generally, a desirable TMM is required to possess high thermal conductivity, good mechanical and chemical stability, excellent electrical insulation, and good processability [7]. Thermally conductive metals such as copper and aluminum are commonly used as TMM, but they suffer from electromagnetic interference [8,9]. Although ceramic thermally conductive materials, such as alumina and silicon nitride, possess excellent thermal conductivity and electrical insulation, their high brittleness poses challenges for their applications [10]. Otherwise, polymer materials provide unique advantages and broad design space for thermal management applications due to their lightweight, electrical insulation, high processability, and decent mechanical properties [11]. Nevertheless, traditional polymers typically exhibit strong phonon scattering and a small mean free path due to their inherent amorphous structure, which can be thermally compared to a combination of many defects [12]. Consequently, most polymers have low intrinsic thermal conductivities, generally ranging between 0.1 and 0.44 W∙m^−^^1^∙K^−^^1^, severely restricting their applications in the high thermal conductivity fields [12]. To address this issue, thermally conductive nanofillers such as carbon black, graphene, metallic fillers, and ceramic fillers were incorporated into the polymer matrix [13]. The key to preparing thermally conductive composites is to realize the effective propagation of phonons. Over the past years, methods such as the percolation threshold method and the construction of segregated network structures have been widely employed to prepare TMMs [13,14,15]. However, both methods make it difficult to escape the drawbacks of using conductive fillers with optimal content or weak interfacial bonding strength [13]. In view of this, aligning fillers in specific directions within the polymer matrix could create an effective thermal conduction path since heat flow conduction can be preferentially carried out along the length direction of the filler [16]. Several tactful strategies have been proposed to realize the filler orientation, such as ice-templated assembly [17], stretching [18], or the application of electric or magnetic fields [19]. However, current fabrication methods often struggle to meet the growing demand for thermally conductive materials with complex shapes. The trend of increasing complexity in modern electronic devices underscores the importance of developing efficient, precise, and shape-specific thermally conductive materials. Therefore, it’s imperative and urgent to develop new approaches to accommodating the production requirements for thermally conductive materials of intricate shapes.

Three-dimensional printing emerged as an enticing manufacturing technology capable of generating a 3D object layer-upon-layer directly from CAD data, regardless of the complexity of the parts to be built. Benefiting from the layer-wise advantages, a variety of functional materials have been fabricated, such as loading-bearing devices [20,21], sensors [22], electronic devices [23,24], and soft actuators [25]. Controlling the condensed structure of materials by shear field, usually derived from extrusion-type 3D printing such as fused deposition modeling (FDM) and direct ink writing (DIW), is acknowledged as one of the important ways to prepare high-performance or multifunctional materials. During the FDM 3D printing process, the pronounced shear field generated from the nozzle promotes the orientation of semi-crystalline polymer molecular chains, accelerating the kinetic process of crystallization and thus yielding superior printed parts. For instance, Yuval Shmueli conducted in-situ measurements on isotactic polypropylene during 3D printing and determined the varied shish–kebab structure resulting from the stretched orientation of the molecular chains caused by the sufficient shear force upon the extrusion process [26]. Similar phenomena have been observed in other studies, some of which even identified hybrid shish-kebab structures that effectively enhanced the mechanical properties of printed parts [27,28]. With respect to the fabrication of the functional parts, an effective method for achieving local control over the structure and thermal performance of composite materials is to strategically align fillers induced by shear-inducing flow to obtain site-specific properties [29]. For example, LLDPE/BN@GNPs composites were printed by FDM with a printing speed of 600 mm/min, and a thermal conductivity of 3.11 W∙m^−^^1^∙K^−^^1^ at 3.51 vol% GNPs content along the printing direction was achieved [30]. The high-efficiency thermal conductivity could be well attributed to the alignment of GNPs and BN subjected to shear and compression forces upon 3D printing. In addition, boron nitride/alumina/polydimethylsiloxane (BN/Al_2_O_3_/PDMS) composites were achieved via shear-induced alignment of DIW with a heterosexual nozzle, which enabled the implementation of large shear forces [31]. The results showed that interconnected thermally conductive networks were constructed by well-aligned BN platelets together with Al_2_O_3_ particles. Therefore, the composites demonstrated an enhanced in-plane thermal conductivity of 3.64 W∙m^−^^1^∙K^−^^1^. While it is well known that fillers could align during extrusion 3D printing, we noted that most of the studies focused on the influence of filler loading on the thermal performance of 3D-printed parts. The shear-induced particle orientation during 3D printing is affected by material components and process parameters such as printing speed, printing temperature, etc. However, unfortunately, previous studies roughly attributed the improvement of thermal conductivity to the effect of shear-induced filler orientation, but some crucial details such as printing speed were often not considered, making the interpretation of the results of 3D printing for the performance improvement of the part limited.

To address the above issue, herein, PDMS/BN slurry suitable for the DIW process was prepared by ultrasound to disperse BN evenly. Then, the BN loadings on the rheological and printing properties of composites were well investigated, indicating that a loading content of 40 wt% BN is favorable. On this basis, the influence of various printing speeds on the orientation degree of BN and the thermal conductivity of composites were studied, respectively. Additionally, the shear field, velocity field, and pressure field at various printing speeds were simulated using Polyflow software to quantify the effect of printing speed on shear field orientation. Results showed that the thermal conductivity of the as-printed parts increased with the increasing printing speed, and PDMS/BN composites finally achieved a thermal conductivity of 0.82 W∙m^−^^1^∙K^−^^1^ with a printing speed of 120 mm/s. This study sheds some light on controlling the performance of TMM with programmed filler orientations.

## 2. Materials and Methods

### 2.1. Materials

BN platelets with an average size of 1–2 μm (Figure S1) were purchased from Shanghai Aladdin Biochemical Technology Co., Ltd. (Shanghai, China), and PDMS precursors (Sylgard 184) were supplied by Dow Corning Corporation, Midland, MI, USA.

### 2.2. Preparation of PDMS/BN Composites

**Figure 1:**
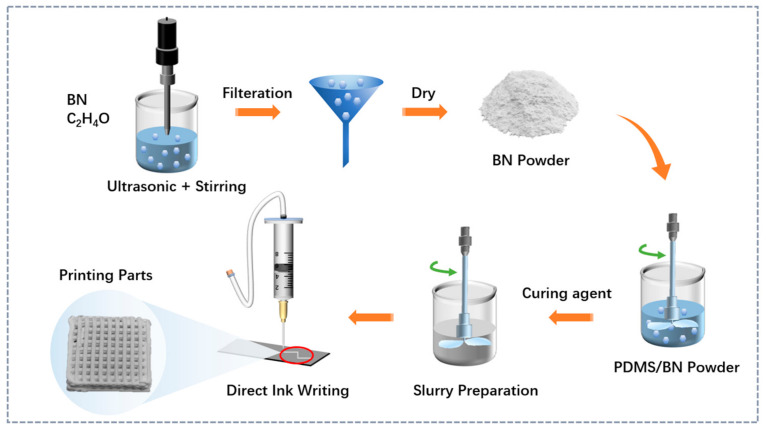
Schematic diagram of preparation procedures of 3D printing PDMS/BN parts.

The preparation process of PDMS/BN composites is schematically shown in Figure 1. To begin with, BN platelets were put into an ethanol solution for 2 h to disperse agglomerated BN particles, followed by filtration, drying, and ball milling. Various BN platelets were mixed with PDMS matrix in an ethanol solution and stirred for 30 min at a stirring speed of 1000 r/min. Subsequently, curing agents were added to the above dispersion in keeping with the need for a mass-quantity ratio of 10:1 and further stirring for 5 min to avoid curing of the compounds at high motion speeds. The obtained compound slurry was then transferred into a barrel of the DIW 3D printer and subjected to mechanical vibration to further remove the bubbles within the slurry. The 3D printing process of PDMS/BN composites was performed on a self-made DIW printer. The corresponding printing models were built by NX 10.0 software and exported as STL files. The slicing process was completed using Simplify3D 4.0 software. The printing parameters were set as follows: nozzle diameter 0.4 mm; infill percentage 100%; printing speed 10, 60, and 120 mm/s; layer thickness 0.3 mm. Our self-made DIW printer employs a 3D motion mechanism based on the XYZ Cartesian coordinate system, in which the printing nozzle moves within the XY plane while the platform moves along the *Z*-axis direction. To achieve high movement precision, a common gantry-type structure is used. Our home-made DIW printer employs pneumatic feeding. In detail, the top of the material cylinder closely matches the rubber valve and is connected to an air compressor via a plastic hose. The air pressure is controlled by the external pressure regulating valve to complete the overall delivery and stable extrusion of the ceramic slurry. In terms of the 3D printing of PDMS/BN composites, the air pressure was well controlled at 0.2 MPa for stable printing. In particular, the molding control group was prepared by pouring the mixed slurry directly into the custom mold. The resultant samples were transferred to a forced-air oven at 80 °C and then cured for 2 h.

### 2.3. Characterizations

The rheological properties of PDMS/BN slurry were measured using a rotational rheometer (MCR 302, Anton Paar, Graz, Austria). The viscosity test was performed at a shear rate range of 0.01–100 s^−^^1^. The frequency sweep test was performed in oscillation mode with a frequency range of 0.1–100 Hz and a temperature of 25 °C. The yield stress was measured under the shear stress sweep mode with a shear stress range of 1–1000 Pa. An X-ray diffractometer (DX-2700 BH, Haoyuan Instrument Co., Ltd., Dandong, China) was used to measure the orientation degree of BN within the PDMS composites using Cu Kα radiation (λ = 0.154 nm) at ambient temperature with a scan range of 10° to 60°. A field emission scanning electron microscope (Hitachi SU8010, Hitachi, Tokyo, Japan) was used to observe the cross-sectional morphology and microstructure of DIW-printed parts. Before SEM observation, the test specimens were subjected to a brittle fracture process in liquid nitrogen, followed by gold coating under vacuum conditions. The thermal conductivity of the samples was based on the transient plane source (TPS) method and detected by using a hot disk thermal constant analyzer (TPS 2200, Hot disk, Gothenburg, Sweden). The thermal conductivity (k) of the samples was calculated according to the following formula κ = α ρ Cp, where α represents thermal diffusivity (in units of m^2^/s), ρ represents the density of the composite (in units of g/cm^3^), and Cp is the specific heat capacity (in units of J/g·K) and measured by using a differential scanning calorimeter (TA-DSC 25, TA Instruments, New Castle, DE, USA). Additionally, the thermal response and thermal management capacity of the samples were evaluated using an infrared thermal imager (FOTRIC 220 RD, FOTRIC, Shanghai, China). Briefly, samples with a diameter of 12.6 mm and a thickness of 3 mm were initially heated to 70 °C and kept for 10 min. Subsequently, the samples were rapidly transferred to an insulating foam, recording the temperature drop time. The volume resistivity of the printed samples was measured using a Keithley 2601B. An Agilent LCR analyzer was employed to evaluate the dielectric constant and dielectric loss.

**Figure 2:**
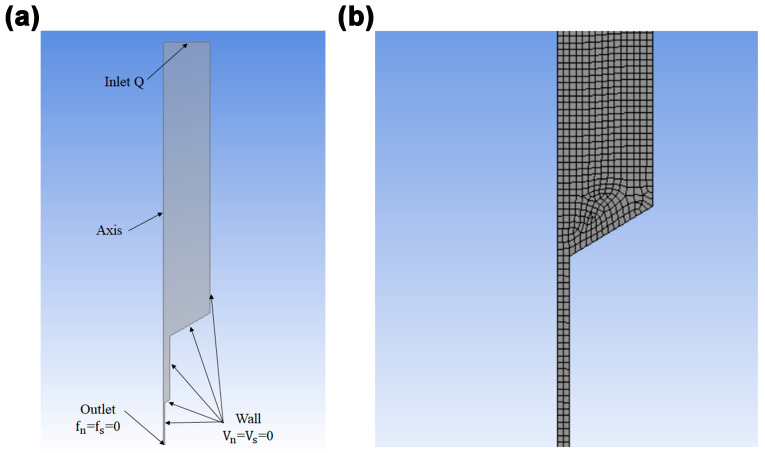
Boundary condition setting (**a**) and meshing (**b**) for the Polyflow simulation of PDMS/BN composites during extrusion of DIW 3D printing.

ANSYS Polyflow19.0 software was used to simulate the rheological properties of slurry in the flow channel to provide a theoretical basis for the 3D printing of PDMS/BN composites. The detailed dimensions of the geometric model for the printing cylinder are shown in Figure S2. To better perform the simulation, the following assumption was made: During the DIW process, the slurry is considered an incompressible and steady-state fluid; the nozzle is fully filled with slurry and retains an isothermal and laminar condition; the inertial and gravitational forces are overlooked; and no slip occurs between the slurry and the internal wall of the nozzle. The flow boundary conditions were set as shown in Figure 2. The inlet volumetric flow rate Q was calculated according to the following formula: $Q=\bar{v}\cdot A$, where *v* is the average velocity and A is the cross-section of the nozzle. Since no wall slip is assumed, and therefore $V_{s}=V_{n}=0\;mm/s$. In addition, in the outlet, the normal force and surface force were all set to zero, namely $f_{n}=f_{s}=0$, as shown in Figure 2a. As for meshing, the element size was 0.1 mm, and the total element number was 76,427.

**Figure 3:**
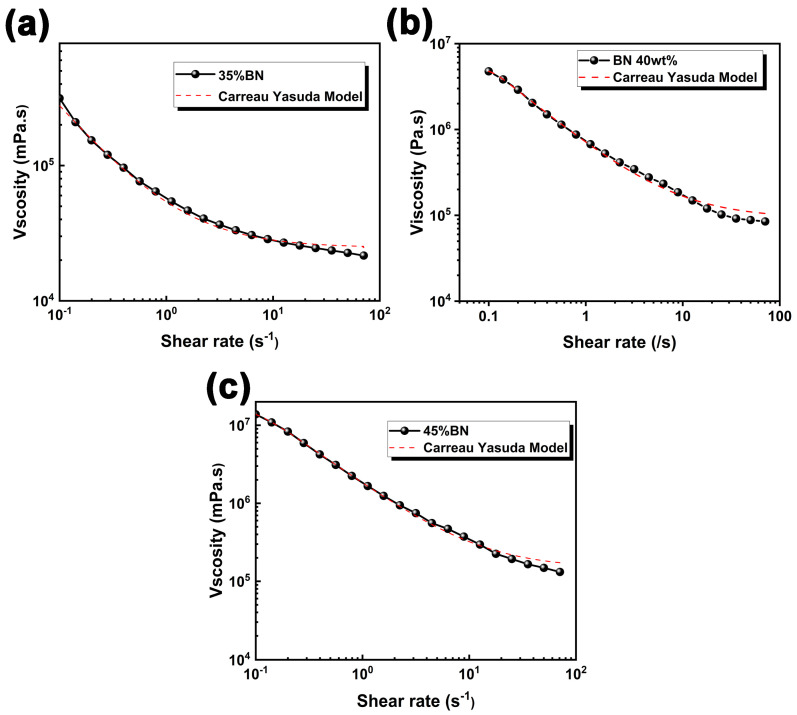
Constitutive model of systems with various BN contents fitted by Carreau–Yasuda model. (**a**) 35 wt% BN; (**b**) 40 wt% BN; (**c**) 45 wt% BN.

The Carreau–Yasuda model is a viscosity model that is widely employed for non-Newtonian fluids, encompassing a variety of systems such as high polymer solutions and suspensions. Based on the above assumptions, the Carreau–Yasuda model is selected as the governing equation and constitutive equation of slurry dynamics. The mathematical expression of the model is as follows:

$$\frac{\eta -\eta_{\infty }}{\eta_{0}-\eta_{\infty }}=\frac{1}{{\left[1+{\left(\lambda \dot{\gamma }\right)}^{a}\right]}^{\frac{1-n}{a}}}$$

where $\eta_{\infty }$ is the ultimate shear viscosity; $\eta_{0}$ is the zero-shear viscosity, λ is the relaxation time, *n* is the power law exponent, and a is a constant controlling the rate of viscosity transition from the zero-shear Newtonian plateau to the shear-thinning region. According to the Carreau–Yasuda model, the viscosity-shear rate curve of various BN loadings was fitted, as shown in Figure 3. For the BN loading-dependent rheological properties, the inlet flow is 1.0028 mm^3^, and the corresponding parameters obtained by fitting the Carreau–Yasuda model are listed as follows, as shown in Table 1.

For the printing speed-dependent rheological properties, three printing speeds were used in the study, specifically 10 mm/s, 60 mm/s, and 120 mm/s, corresponding to volumetric flow rates of 4.752 mm^3^/s, 2.376 mm^3^/s, and 0.396 mm^3^/s, respectively.

## 3. Results

### 3.1. Evaluation of Rheological Behavior during DIW Printing

During the DIW process, the ink initially flows at a notably low velocity within the syringe and then rapidly accelerates to a high shear rate when flowing through the nozzle. After being extruded from the nozzle, the ink is deposited on the platform and returns to a state of rest. As an extrusion-based 3D printing technology, the printing quality and efficiency of DIW 3D printing greatly depend on the rheological properties of the slurry. Therefore, the rheological behavior of the flowable slurry is critical, as it will determine the extrusion process and the behavior of the filament at the deposition state.

**Figure 4:**
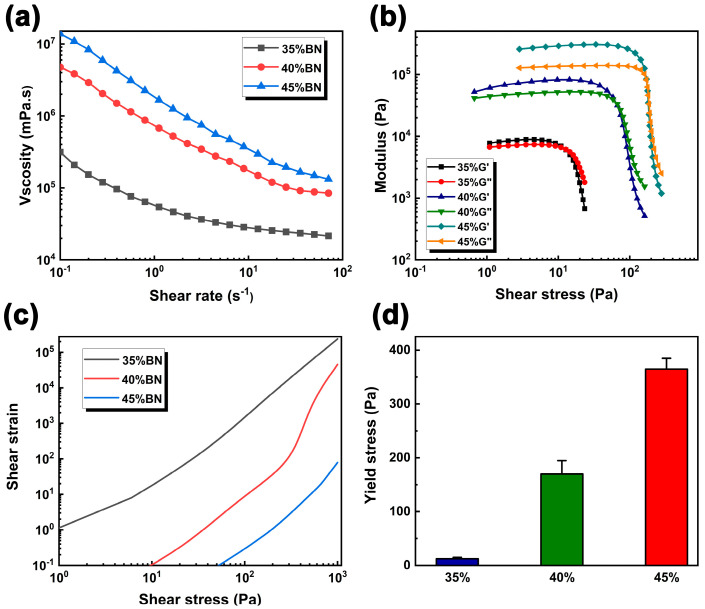
The rheological properties of PDMS/BN slurry; (**a**) viscosity as a function of shear rate with various BN loadings; (**b**) storage modulus G′ and loss modulus G″ as a function of oscillatory stress; (**c**) deformation as a function of shear stress; (**d**) the corresponding yield stress value from (**c**).

The rheological properties of the PDMS/BN composite slurry are presented in Figure 4. As shown in Figure 4a, the viscosity of the composite slurry decreased with an increase in the shear rate at a constant BN loading, exhibiting a “shear thinning” behavior. This characteristic is conducive to the consistent flow of the slurry from the nozzle since an implication of the decrease in viscosity with increasing shear rate is the remarkable reduction of the extrusion pressure. In addition, as the BN content increased, the viscosity of the system also increased rapidly. This can be well attributed to the interaction within the composites as well as the network structure formed by BN particles, which enhanced the overall viscosity of the system. Figure 4b displays the viscoelastic properties of the composite slurry. As shown, the storage modulus (G′) and loss modulus (G″) of the system remained almost unchanged. It is well known that G′ represents the elasticity of the material, while G″ characterizes the viscosity of the material. In the linear viscoelastic region (LVER), the value of G′ was higher than that of G″, indicating a solid-like behavior with mainly elastic behavior, which could maintain the structural stability of the composites. Notably, the LVER of the composite slurry increased with the increasing BN loadings because the resulting network structure enhanced the system’s resistance to external strain, thereby maintaining its linear viscoelastic behavior over a wider strain range. With the increase in shear stress, both G′ and G″ decreased rapidly, and the value of G″ was still higher than that of G′, suggesting solid-like behavior with irreversible deformation. When the crossover of the curves was reached, G′ was equal to G″, indicating a viscoelastic transition from solid-like behavior to liquid-like behavior. This is because shear-induced relative displacement between BN particles starts disrupting the elastic network structure of the slurry, resulting in viscosity-dominated and shear-thinning properties [32]. We further measured the yield stress of the composite paste to evaluate its shape stability after printing, as shown in Figure 4c. The whole curve can be roughly divided into two stages. With the increase in shear stress, the initial deformation increases slowly, and the internal network structure of the system remains intact at this stage. However, with further elevation in shear stress, the deformation increased rapidly, implying the onset of network disintegration. The yield stress was determined from the turning point of the curve, and the corresponding results are displayed in Figure 4d. The yield stress increased from 9.6 Pa to 364 Pa as the BN loading ranged from 30 wt% to 45 wt%, indicating improved structural stability when subjected to greater stress. Note that the yield stress can also be determined from the crossover point of the G′ and G″ curves in Figure 4c. The yield stress values obtained by the two methods may differ since the shear strain–shear stress test is a steady-state rheological mode while the frequency sweep is an oscillatory mode.

**Figure 5:**
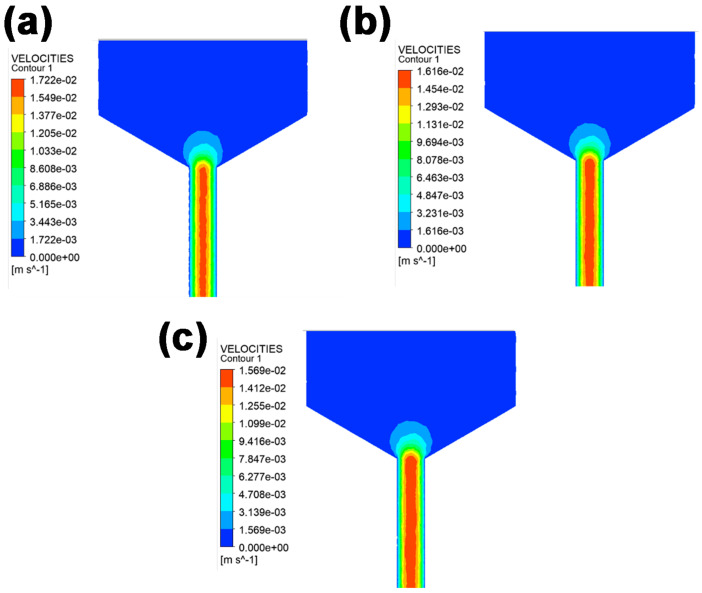
Velocity distribution in the flow field of PDMS/BN composites with various BN loadings. (**a**) 35 wt% BN, (**b**) 40 wt% BN, and (**c**) 45 wt% BN.

To provide deep insight into the influence of BN content on the rheological properties of composite slurry, Polyflow software was employed to study the extrusion process within the syringe. The flow field distribution directly reflects the velocity, shear, and pressure fields experienced by the BN filler during the extrusion process. This significantly affects the final ordered structure and distribution of BN filler, thereby influencing the thermal conductivity of the fabricated parts. The velocity field was studied, as shown in Figure 5. One can see that the velocity of the printing material within the barrel was relatively low, almost close to zero in speed. However, as the composite slurry approached the convergence areas, a gradual increase in velocity was observed. Upon entering the slender printing nozzle with a diameter of 0.4 mm, the composite slurry exhibited a marked gradient distribution in velocity. The highest velocity was detected close to the central axis, while the lowest velocity was observed adjacent to the barrel wall. Moreover, the velocity field distribution of the extruded paste showed a distinct dependency on the content of BN filler. The maximum extrusion velocity decreased with increasing BN content. For instance, the maximum velocity at the central axis reached 1.722 × 10^−2^ m/s when the BN content was 35 wt%. However, when the BN content increased to 45%, the velocity at the central axis faded to just 1.569 × 10^−2^ m/s.

**Figure 6:**
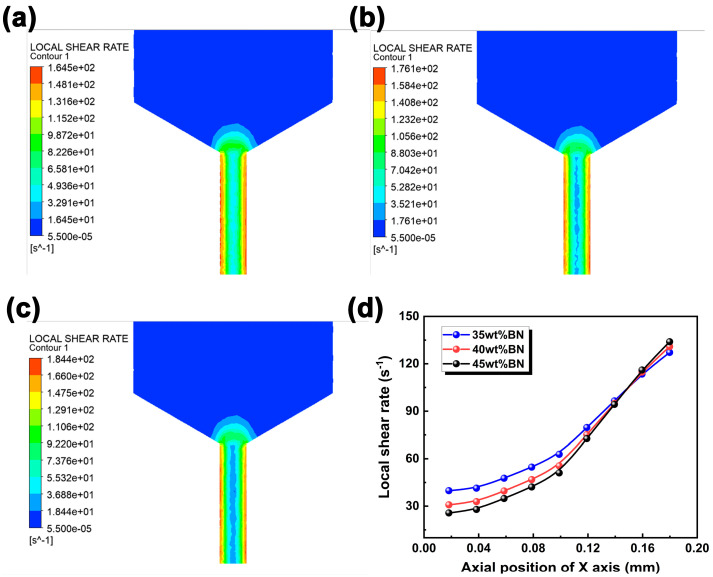
Local shear rate in the flow field of PDMS/BN composites with various BN loadings. (**a**) 35 wt% BN, (**b**) 40 wt% BN, (**c**) 45 wt% BN, and (**d**) the shear rate distribution along the radial direction of the printing nozzle under different BN loadings.

The orientation and distribution of BN fillers were impressively influenced by the distribution of the shear rate during the DIW printing process, thus affecting the thermal conductivity of the final printed parts [29]. As presented in Figure 6, the local shear rate within the barrel was analyzed. For a specific BN content, the shear rate distribution exhibited the opposite law to the velocity distribution. Lower shear rates were found near the central axis of the nozzle, whereas higher shear rates were observed closer to the nozzle wall, forming a typical shear rate gradient. In addition, the shear rate inside the barrel showed a significant difference, exhibiting a complex pattern. Specifically, near the central axis of the nozzle, the composite slurry with 35% BN showed the highest shear rate. However, near the nozzle wall, the composite slurry with 45% BN demonstrated the highest shear rate. Indeed, the PDMS/BN system is a non-Newtonian fluid, so the Herschel–Bulkley model is suitable to describe its rheological behavior. At a lower content, the shear stress was responsible for the rheological behavior of the PDMS/BN composite slurry. The internal resistance of the slurry was reduced due to a lower yield stress, thus leading to a higher local shear rate with 35 wt% BN. On the other hand, with a 45% BN content, the rheological behavior of the composite slurry was primarily influenced by the concentration of BN particles. The yield stress was elevated due to the increasing BN content, thereby heightening the internal resistance. In this situation, even though the shear stress is smaller near the wall, the shear rate could remain high because of the increase in internal resistance. As a result, a higher local shear rate of approximately 184 s^−1^ was achieved at 45 wt% BN content, higher than that of 164 s^−1^ for 35 wt% BN content.

Rheological results showed that the yield stress of composite slurry increased with the increase in BN content, which helped to maintain the shape and structural stability of printed parts. Nevertheless, this also resulted in a significant increase in apparent viscosity, complicating the extrusion process. Our research goal is to alter the orientation of BN by adjusting the shear rate under minimized BN content, thereby constructing a conductive path. Therefore, the thermally conductive filler content should not be excessively increased. Considering the balance between the printability and thermal conductivity of the printed components, we chose a BN content of 40% as the optimal study focus.

### 3.2. Effect of Printing Speed on the Orientation of BN Particles during DIW Printing

**Figure 7:**
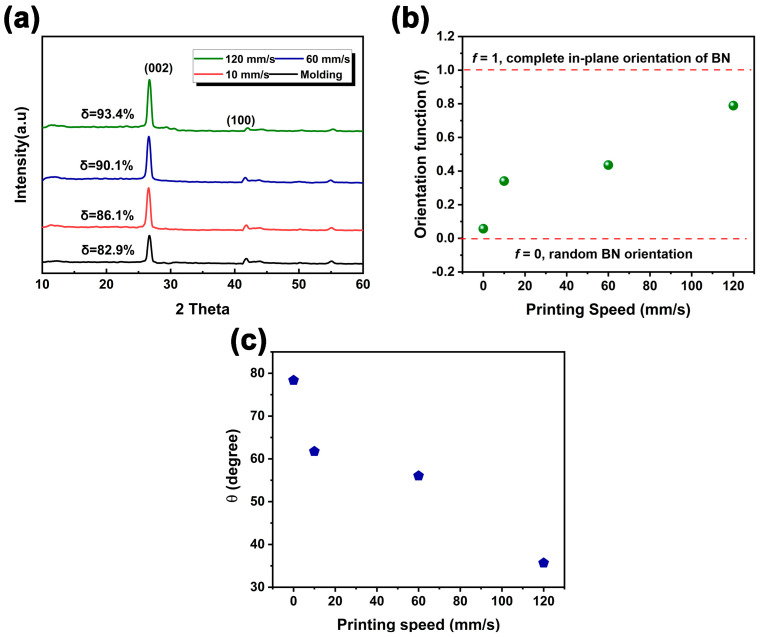
(**a**) XRD patterns, (**b**) orientation function, and (**c**) orientation angle of PDMS/BN printed parts with various BN loadings ranging from 35 wt% to 45 wt%.

Printing speed can have a significant impact on the orientation of BN particles during the DIW printing process because the composite slurry experiences high shear forces. Accordingly, the effect of three printing speeds (10, 60, and 120 mm/s) on the orientation structure of BN particles during the DIW extrusion process was investigated. XRD has been commonly considered an effective way to characterize the orientation of BN platelets in composites [33]. Figure 7a presents the XRD spectra for PDMS/BN printed parts at various printing speeds. PDMS is an amorphous polymer, so all the diffraction peaks that appeared in the XRD pattern can be attributed to the crystal planes of BN platelets. Three diffraction peaks were observed, located at 26.9°, 41.6°, and 55.1°, corresponding to the (200), (100), and (004) lattice planes, respectively. As can be seen, with the increasing printing speed, the intensity of the (200) lattice plane gradually increased, while that of the (100) lattice plane slightly weakened. In the crystal structure of BN, the (002) and (100) lattice planes represent the interlayer and in-plane directions, respectively. Considering that BN is anisotropic, an increased intensity of the (002) peak indicates a greater degree of alignment of BN platelets along the c-axis, which is perpendicular to the interlayer direction or “out-of-plane”. This suggests the enhanced orientation of BN platelets in a direction vertical to the surface of the printed parts. Conversely, the intensity of the (100) peak reflects the orientation of BN platelets within the layers, or “in-plane”, indicating the extent of BN alignment in a direction horizontal to the surface of the printed parts. The intensity ratio of (002) to (100) increased with the increasing printing speed when measured from the top surface of the printed parts, indicating enhanced BN alignment along the horizontal direction. For more quantitative analysis, an orientation degree (δ) was used and defined as Equation (1).

(1) $$\delta =\frac{I_{200}}{I_{200+I_{100}}}$$

where *I*_(100)_ and *I*_(002)_ represent the intensity of the (100) and (200) lattice planes, respectively. As shown in Figure 7a, as the printing speed increased, the value of δ increased from 82.9% of the control sample to 93.4% of the 120 mm/s sample. A higher δ was obtained at a higher printing speed, suggesting that more BN platelets were aligned along the horizontal direction. Furthermore, the orientation function *f*, defined as Equation (2), was used to describe the orientation degree of the BN platelet *c*-axis relative to the *z*-axis composite slurry [34].

(2) $$f=\frac{1-K}{1+2K}$$

(3) $$K=\frac{k\cdot I_{100}}{I_{200}}$$

where *k* is the normalization coefficient and is assigned a value of 6.25 according to the intensity ratio of (002) to (100). A value of *f* = 1 indicates complete alignment of the BN platelets along the horizontal direction. Conversely, *f* = −0.5 denotes the BN platelets along the vertical direction. Otherwise, *f* = 0 implies a random distribution of BN platelets within the PDMS matrix. The orientation function *f* of PDMS/BN printed parts under three different printing speeds was calculated, as shown in Figure 7b. Three-dimensionally printed parts at a printing speed of 120 mm/s presented an *f* value of 0.79, indicating a high degree of BN along the horizontal direction. On the other hand, the *f* value of the control sample was almost close to 0, indicating a random orientation state. Moreover, the relationship between the orientation angle θ and the orientation degree *f* can be established with the following formula:

$$f=\frac{\;(3<\cos\theta >-1)}{2}$$

Accordingly, the orientation degrees under various printing speeds were calculated, as shown in Figure 7c. For the control sample, an orientation degree of 78.3° indicates a random orientation state within the molding-processed sample. One can see that the orientation degree was reduced from 61.7° to 35.7° as the printing speed ranged from 10 mm/s to 120 mm/s, indicating that more BN platelets were aligned in the horizontal direction, which is also the printing direction. This can be well explained by the fact that a high shear force was applied to the BN platelets under the high printing speed, thereby facilitating BN particle flipping and eventual alignment along the printing direction.

**Figure 8:**
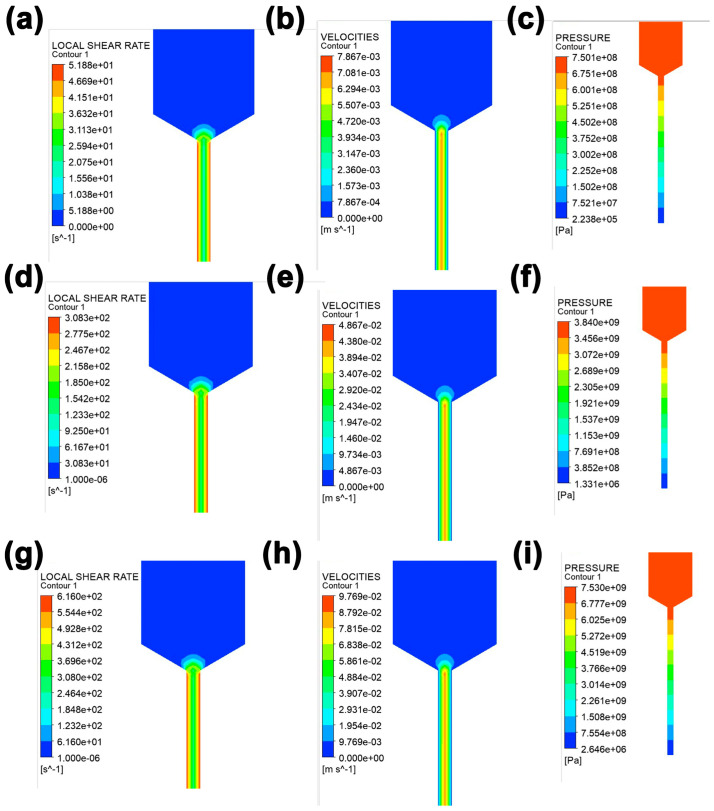
Local shear rate (**a**,**d**,**g**), velocity (**b**,**e**,**h**), and pressure (**c**,**f**,**i**) in the flow field of PDMS/BN composites at various printing speeds of 10 mm/s (**a**–**c**), 60 mm/s (**d**–**f**), and 120 mm/s (**g**–**i**).

To obtain a deep insight into the effect of printing speed on the orientation of BN particles, Polyflow software was used, and the shear rate distribution, velocity field distribution, and pressure field distribution at different printing speeds were obtained, as shown in Figure 8 and Figure S3. Considering that PDMS/BN composite slurry is a non-Newtonian fluid, the shear rate γ can be defined with the formula γ = $\frac{(3n+1)Q}{{\pi R}^{3}}$, where Q is the volume flow rate, R is the nozzle radius, and n is the behavior index of the non-Newtonian fluid. Therefore, high printing speed results in a large Q, leading to an overall higher shear rate. As the printing speed increased, the maximum shear rate increased from approximately 52 s^−1^ to 616 s^−1^ (the detailed values of the shear field distribution along the radial direction of the nozzle are shown in Figure S3), indicating that the BN particles would experience a greater shear force at high printing speeds. The increased shear force facilitated the movement and rotation of BN particles, which were easier to align along the printing direction. In addition, a high shear rate would decrease the viscosity, thereby assisting the printability of the composite slurry. Furthermore, to determine the flow characteristics of the composite slurry through the nozzle diameter, the Reynolds number (*R*_e_) was calculated using Equation (4) [35]:

(4) $$R_{e}=\frac{\rho \nu L}{\mu }$$

where ρ is the density of the slurry, ν is the velocity, L is the characteristic length of the nozzle, and μ is the dynamic viscosity. Utilizing a viscosity of μ = 6.74 × 10^5^ Pa.s (viscosity of 40 wt% BN at 1 rad/s), ρ = 1.3 g/cm^3^, ν = 10, 60, and 120 mm/s, and L = 0.4 mm, *Re* was calculated to be 7.7 × 10^−7^, 4.6 × 10^−5^, and 9.2 × 10^−5^, respectively. Generally, the *Re* is commonly employed to distinguish various flow regimes in pipes. Specifically, laminar flow is represented by *R_e_* ≤ 2000, critical flow corresponds to 2000 < *R*_e_ < 4000, and turbulent flow is indicated when *R_e_* > 4000. The determined *R_e_* for various printing speeds is much smaller than the critical threshold of 2000, suggesting a laminar flow during the DIW extrusion process [36]. Due to the uniform and continuous shear force generated in the laminar flow, BN particles were arranged according to the flow direction at a higher printing speed, constructing a microstructure with an ordered orientation and thus enhancing the thermal conductivity of the printed parts.

### 3.3. Morphologies Observation of the Printed PDMS/BN Composites

**Figure 9:**
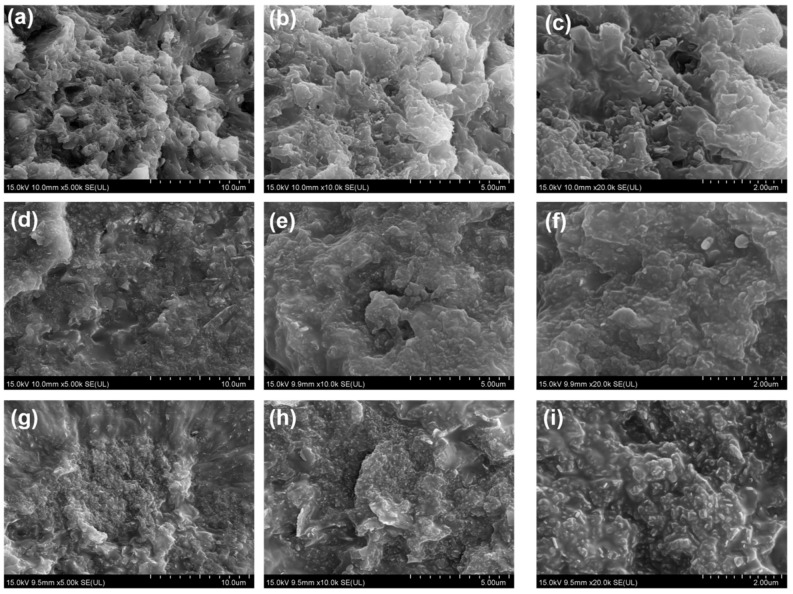
SEM images of 3D-printed BN/PDMS parts with various printing speeds of 10 mm/s (**a**,**d**,**g**), 60 mm/s (**b**,**e**,**h**), and 120 mm/s (**c**,**f**,**i**).

In order to further understand how the printing speed affects the microstructure and properties of BN/PDMS printed parts, SEM observations were performed on the samples prepared at various printing speeds. Figure 9 displays the SEM images of fractured cross-sections of the printed parts. The cross-sectional morphology of printed parts at different printing speeds presents completely different situations. At a lower printing speed of 10 mm/s, many small white bumps were observed on the cross-section of the printed part, which could well be attributed to the flexible PDMS matrix. Only a small amount of BN particles can be discerned on the cross-section, and most of the BN particles were completely wrapped by PDMS. This is because BN particles can still sustain a uniform and irregular distribution within the PDMS matrix when subjected to a small shear stress during low-speed printing. Under such conditions, BN particles were robustly wrapped by PDMS, making it challenging to be observed. In contrast, as the printing speed increased, especially when it reached 120 mm/s, the cross-sectional morphology changed remarkably. An abundance of BN particles was exposed, and the a-axis of many BN particles points out of the section, indicating that these BN particles are oriented in the printing direction. BN particles are easily affected by shear force during the DIW extrusion process due to their inherent flat sheet structure. As can be seen in Figure 8, the slurry experienced a boost in shear rate from 52 s^−1^ to 616 s^−1^ as the printing speed increased from 10 mm/s to 120 mm/s. The sheet-like BN particles then tended to flip themselves to align the a-axis direction with the flow direction (i.e., the printing direction) to minimize the flow resistance. Subsequently, during the applied shear flow, the as-flipped BN particles would translate along the printing direction and finally align along the printing direction. Based on this, the aligned BN particles can construct an effective thermal conduction path, which is helpful to improve the thermal conductivity of the resultant parts.

### 3.4. Thermal Conductivity Performance of the as-Printed PDMS/BN Composites

**Figure 10:**
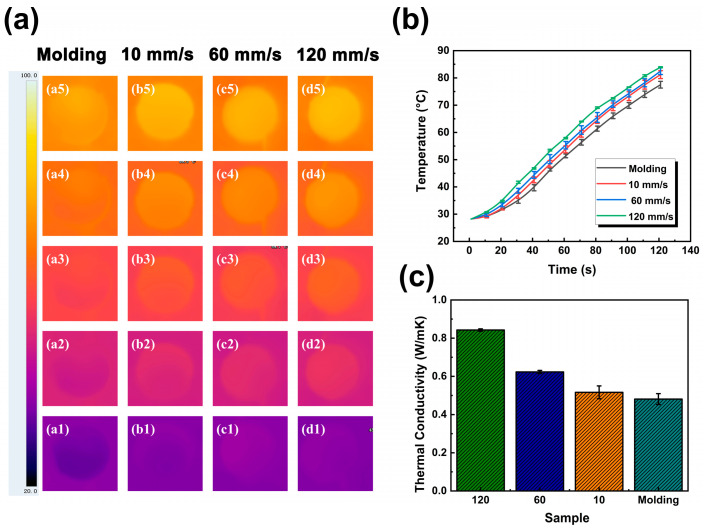
Thermal conductivity performance of 3D-printed samples with various printing speeds (10, 60, and 120 mm/s), and the control sample. (**a**) Optical IR images taken every 20 s within 120 s. a1–a5 represent molding sample. b1–b5, c1–c5, and d1–d5 represent samples with a printing speed of 10, 60, and 120 mm/s, respectively. (**b**) The recorded surface temperature curves during the heating process. (**c**) Thermal conductivity.

To study the capability of thermal conductivity printed parts, the temperature of surface variation upon the heating process was measured using IR thermal images. To ensure the accuracy of the test, the samples of the resultant PDMS/BN printed parts and the random molding sample have the same dimensions and thickness. The recorded thermal images are shown in Figure 10a, and the corresponding surface temperatures of the tested samples over time were inspected and plotted in Figure 10b. All the samples started the heating process at room temperature. As can be seen, in the initial stage of heating within 10 s, the temperature of all samples changed relatively slowly, which may be related to the heat conduction characteristics inside the sample, and it required a certain amount of time for the heat to be evenly distributed. Subsequently, the temperature gradually increased with an almost linear relationship with time, reflecting the continuity and uniformity of the heat conduction path inside the sample. In addition, the temperature of 3D-printed samples was generally higher than that of the control sample during the whole test period, highlighting the better thermal conductivity of 3D-printed samples compared with the molding sample. Moreover, as the printing speed increased, the surface temperature of the sample also increased accordingly. Particularly, the average surface temperature of the sample with a printing speed of 120 mm/s was approximately 7 °C higher than that of the control sample. This can be well attributed to the alignment of BN particles along the printing direction during the 3D printing process, thus constructing more efficient heat conduction channels.

The in-plane thermal conductivity of 3D-printed PDMS/BN composites with various printing speeds is shown in Figure 10c. It was reported that the thermal conductivity of pure PDMS was about 0.185 W∙m^−1^∙K^−1^ due to the amorphous structure. One can see that with the addition of BN particles, the thermal conductivity of the control sample increased to approximately 0.454 W∙m^−1^∙K^−1^ at 40 wt% BN loading. At a printing speed of 10 mm/s, the thermal conductivity of the 3D-printed composites was almost comparable to that of the control sample. However, the thermal conductivity of the printed part increased continuously to 0.849 W∙m^−1^∙K^−1^ as the printing speed increased, an increase of 87%. This indicated that thermal transport paths were successfully constructed within the composites at high printing speeds.

**Figure 11:**
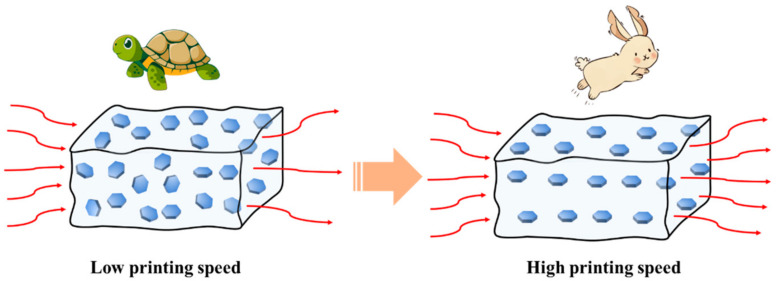
Schematic diagram of heat conduction channel under different printing speeds.

In order to have a more comprehensive understanding of the mechanism of the influence of printing speed on the thermal conductivity of the composites, the Agari equation was applied to determine the effective thermally conductive chains. The Agari equation can be defined as Equation (5) [37,38],

(5) $$LogK_{c}=\varphi C_{2}logK_{f}+(1-\varphi )log(C_{1}K_{m})$$

where K_c_, K_f_, and K_m_ are the thermal conductivities of the composites, fillers, and polymer matrix. K_f_ and K_m_ could be determined as ~300 and 0.185 W∙m^−1^∙K^−1^ according to the previous [37,39]. φ was calculated as 18.98%, 22.06%, and 26.01%, respectively, according to the mass fraction and density of the system. The constant factors C_1_ and C_2_ are related to the specific structure and composition of the composite material and include factors such as filler shape, size, and distribution. Considering the amorphous structure of PDMS, C_1_ was set to be 1. By transforming the equation, the C_2_ value can be used to evaluate the effective thermally conductive chains within PDMS/BN composites with Equation (6).

(6) $$C_{2}=\frac{logk_{c}-\left(1-\emptyset \right)logk_{m}}{\emptyset logk_{f}}$$

where the K_c_ values of four samples were 0.849, 0.624, 0.554, and 0.454 W∙m^−1^∙K^−1^, as presented in Figure 10c. Accordingly, the corresponding values of C_2_ were calculated to be ~1.157, 0.929, 0.858, and 0.732, respectively. As can be seen, the value of C_2_ tended to be higher with increasing printing speed, inferring that more effective thermal conduction channels were formed, benefiting from the high shear rate of 3D printing, as presented in Figure 11. Otherwise, effective thermal conduction paths were difficult to establish in the control sample with the randomly dispersed BN particles, resulting in the dissipation of thermal energy. These results are in line with the analysis of XRD and SEM.

Furthermore, the electric and dielectric performance of the printed PDMS/BN composites was evaluated, as shown in Figure S4. One can see that the printing speed has little influence on the resistivity of the printed composites, presenting an approximate electrical conductivity of 5.0 × 10^−13^ Ω^−1^·m^−1^. The dielectric constant of all the printed samples remained almost stable with the increase in frequency from 10^2^ to 10^6^ Hz. In addition, the dielectric constant of the PDMS/BN composites exhibited an observable decrease as the printing speed increased, which may have resulted from the enhanced orientation of BN particles leading to their increased alignment or overlapping, thereby reducing the effective interfacial area with the PDMS matrix and consequently reducing the interfacial polarization and the dielectric constant [40]. The dielectric loss decreased with the increase in frequency and remains within 0.16, but there is a small increase in the range of 100–1000 Hz, which may be related to the dielectric relaxation caused by the orientation structure of BN particles. Overall, the printed composites displayed high volume resistivity, a low dielectric constant, and a low dielectric loss, which is suitable for use in electronic devices.

### 3.5. Preparation of Customizable Complex Heat-Conducting Parts Based on 3D Printing

**Figure 12:**
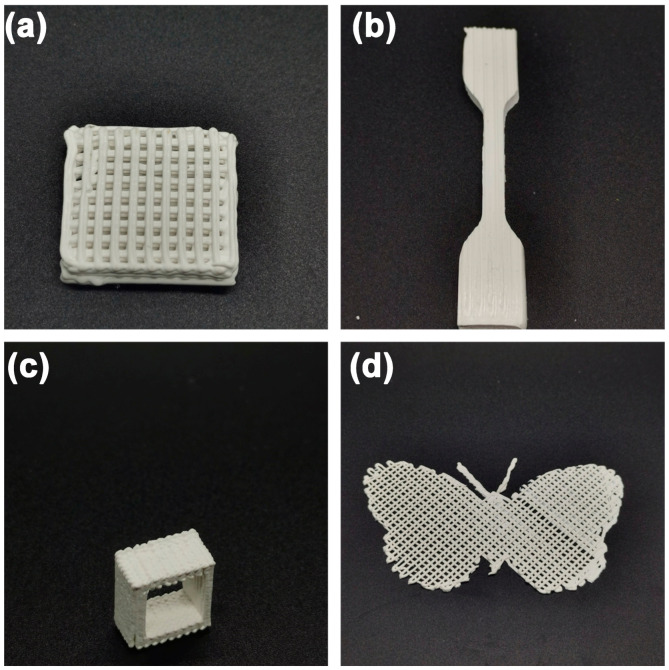
A variety of thermally conductive models fabricated by DIW 3D printing, (**a**) thermally conductive scaffold, (**b**) splines, (**c**) heat dissipation ring, and (**d**) butterfly-shaped complex parts.

Based on the previous discussion, the orientation degree of BN particles in the printed part can be effectively controlled by adjusting the printing speed, thereby regulating the thermal conductivity of the printed part. DIW 3D printing offers a versatile platform for advanced manufacturing due to its convenience, mild printing conditions, and ability to fabricate complex-shaped parts. Accordingly, the thermally conductive scaffold, splines, heat dissipation, and butterfly-shaped complex parts were fabricated, as shown in Figure 12. It should be noted that when printing parts with complex structures, it is crucial to reasonably analyze the main heat flow direction and heat load domains of the component. Ensure that the print trajectory is optimized to harness the full potential of the shear-induced thermal filler orientation inherent in extrusion-based 3D printing. Ultimately, the fabrication of components with enhanced thermal conductivity, specifically designed for pragmatic applications, is realized. In this way, thermal management parts suitable for various heat conduction occasions can be customized through structural design and printing speed regulation.

## 4. Conclusions

In summary, we successfully fabricated PDMS/BN composites by using DIW 3D printing. According to the rheological properties of composites with various BN loadings, an optimal BN loading of 40 wt% was selected to better balance the structural stability of 3D printed parts and the smooth extrusion process. Furthermore, the influence of printing speeds on the thermal conductivity of printed parts was deeply investigated. The results showed that increasing printing speed from 10 mm/s to 120 mm/s raised thermal conductivity from 0.454 to 0.849 W∙m^−^^1^∙K^−^^1^. XRD results showed that the diffraction peak intensity ratio of (002)**/**(100) increased as the printing speed ranged from 10 mm/s to 120 mm/s, and the calculated orientation angle decreased from 78.3° of the comparative sample to 35.7° of 120 mm/s. SEM observations supported the XRD results by showing that many BN particles were exposed and aligned along the printing direction at a printing speed of 120 mm/s. This is because BN particles experienced an increase in shear rate from 52 s^−^^1^ to 616 s^−^^1^, corresponding to an increase in printing speed from 10 to 120 mm/s, as evidenced by Polyflow analysis. All this evidence can strongly prove that the increase in shear rate helps to promote the ordered orientation of BN particles and form thermal conduction channels, thereby improving thermal conductivity. Combined with the advantages of DIW processing, various printed parts with different shapes and thermal conductivity can be customized, shedding some light on the preparation of thermal management devices.

## Figures and Tables

**Table 1:** Corresponding parameters obtained by fitting the Carreau–Yasuda model.

BN Content	$\eta_{\infty }$	$\eta_{0}$	λ	a	n
35 wt%	2.4 × 10^4^	9.53 × 10^5^	40.2	52.6	6.40 × 10^−2^
40 wt%	9.27 × 10^4^	5.0 × 10^6^	9.4	7.96	5.61 × 10^−2^
45 wt%	1.47 × 10^5^	1.80 × 10^7^	11.0	3.10	1.57 × 10^−2^

## Data Availability

Not applicable.

## Supplementary Materials

The following supporting information can be downloaded at: https://www.mdpi.com/article/10.3390/polym15163489/s1, Figure S1. SEM image of BN two-dimensional nanosheet; Figure S2. DIW barrel geometry for POLYFLOW simulations; Figure S3. The detailed values of the shear field distribution along the radial direction of the nozzle; Figure S4. Electrical and dielectric properties of the printed PDMS/BN composites with various speeds. a) volume resistivity, b) dielectric constant, and c) dielectric loss.
