# Supplementary material for: Controlling Shear Rate for Designable Thermal Conductivity in Direct Ink Printing of Polydimethylsiloxane/Boron Nitride Composites
Source: Polymers (Basel). 2023 Aug 21;15(16):3489. doi: 10.3390/polym15163489 (PMC10459169; doi:10.3390/polym15163489)
Supplement: Supplementary file 1 [file polymers-15-03489-s001.zip › polymers-2566029-supplementary.pdf]

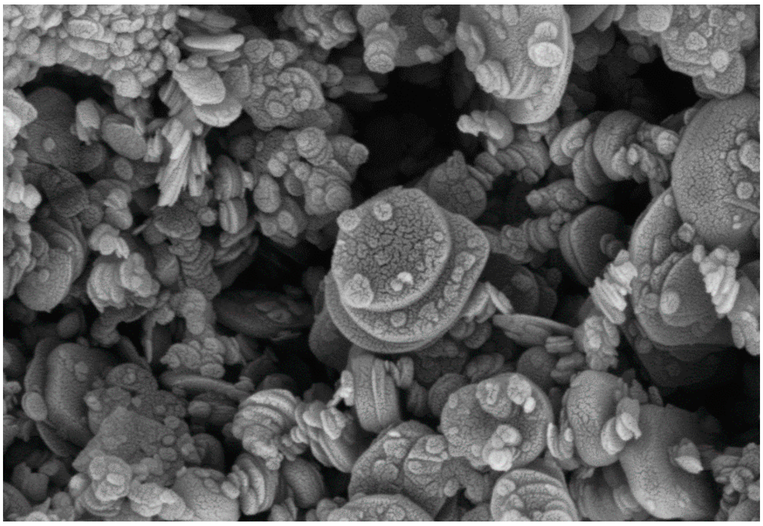

Figure S1. SEM image of BN two-dimensional nanosheet.

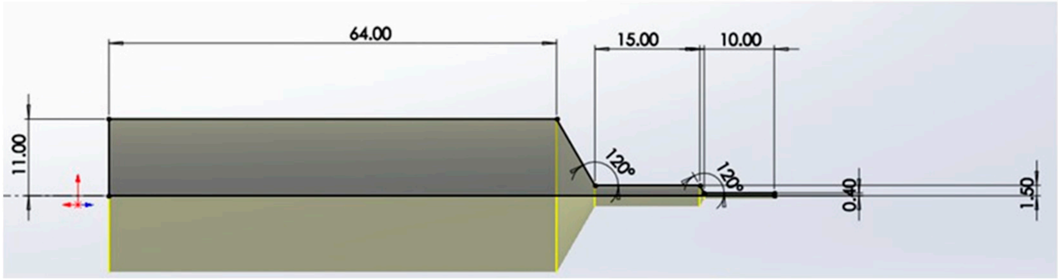

Figure S2. DIW barrel geometry for POLYFLOW simulations.

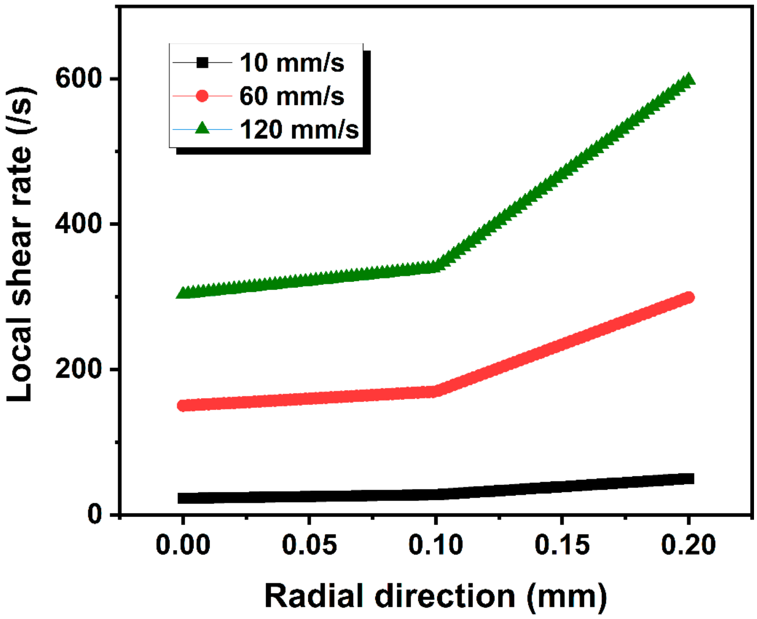

Figure S3. The detailed values of the shear field distribution along the radial direction of the nozzle.

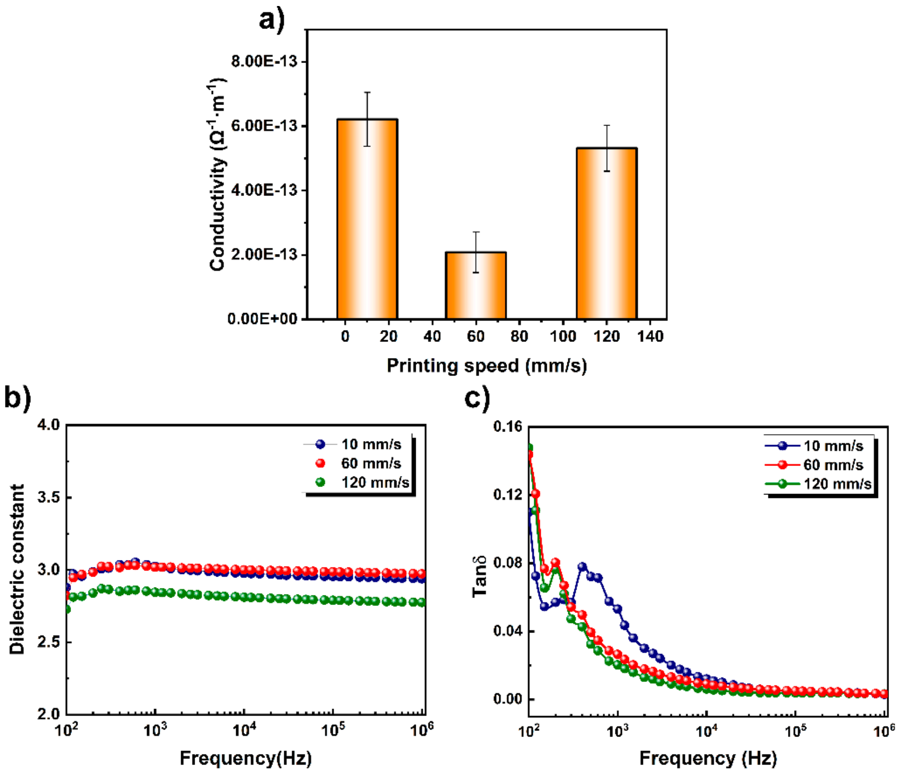

Figure S4. Electrical and dielectric properties of the printed PDMS/BN composites with various speeds. a) volume resistivity, b) dielectric constant, and c) dielectric loss.
